# Involvement and targeted intervention of benzo(a)pyrene-regulated apoptosis related proteome modification and muti-drug resistance in hepatocellular carcinoma

**DOI:** 10.1038/s41419-023-05771-7

**Published:** 2023-04-12

**Authors:** Ye Yang, Ming Jin, Yajie Meng, Yi Dai, Shuai Chen, Yan Zhou, Yuan Li, Liming Tang

**Affiliations:** 1 The Key Laboratory of Modern Toxicology, Ministry of Education, School of Public Health, Nanjing Medical University, Nanjing, 211166 China; 2 The Affiliated Changzhou Second People’s Hospital of Nanjing Medical University, Changzhou, 213003 China

**Keywords:** Cancer, Cancer microenvironment

## Abstract

During the development of hepatocellular carcinoma (HCC), the mutual adaptation and interaction of HCC cells and the microenvironment play an important role. Benzo(a)pyrene (B[a]P) is a common environmental pollutant, which can induce the initiation of various malignant tumors, including HCC. However, the effects of B[a]P exposure on progression of HCC and the potential mechanisms remains largely uninvestigated. Here we found that, after the long-term exposure of HCC cells to low dose of B[a]P, it activated glucose-regulated protein 75 (GRP75), which then induced a modification of apoptosis-related proteome. Among them, we identified the X-linked inhibitor of apoptosis protein (XIAP) as a key downstream factor. XIAP further blocked the caspase cascade activation and promoted the acquisition of the anti-apoptosis abilities, ultimately leading to multi-drug resistance (MDR) in HCC. Furthermore, the abovementioned effects were markedly attenuated when we inhibited GRP75 by using 3,4-dihydroxycinnamic acid (caffeic acid, CaA). Collectively, our present study revealed the effects of B[a]P exposure on the progression of HCC, and identified GRP75 was a meaningful factor involved in.

## Introduction

Benzo(a)pyrene (B[a]P) is a common environmental pollutant, which is usually regarded as an indicator compound of polycyclic aromatic hydrocarbons (PAHs), and classified as a Class I carcinogen [1]. It mainly comes from the incomplete combustion of organic materials and it is also commonly found in high-temperature processed foods, contaminated water, air, and tobacco [2]. In terms of the impact on tumors, B[a]P can induce the development of various malignant tumors. Among them, the liver is an important target organ for the toxic effect of B[a]P, as it possesses a high abundance of cytochrome P450 enzymes, making B[a]P with high retention and low clearance in the liver [3]. After B[a]P enters the liver, it is metabolized to B[a]P-7,8-diol-9,10- epoxide, which is then covalently bound to DNA to form adducts, giving rise to the genotoxic damage of DNA [4]. Meanwhile, intrahepatic B[a]P also induces the generation of reactive oxygen species (ROS), which cause the oxidative stress reaction, phosphorylation modification, and other biological processes, leading to the apoptosis, autophagy, abnormal liver fat metabolism, and hepatocarcinogenesis [5, 6].

Hepatocellular carcinoma (HCC) is the most common malignancy and the third leading cause of cancer-related mortalities globally [7]. For the insidious onset, most patients are in the progressive/advanced stages at the time of definitive diagnosis, so it is always accompanied by poor prognosis [8]. During the development of HCC, the mutual adaptation and interaction of HCC cells and the microenvironment play an important role [9]. On one hand, the surrounding blood vessels, the extracellular matrix, non-malignant cells, cytokines, and other bioactive molecules, together constitute the tumor biological microenvironment, which in turn affect tumor cell growth, and ultimately accelerating the progress of HCC [10]. On the other hand, HCC progress is also greatly influenced by a variety of exogenous factors via forming extracellular chemical microenvironment, including environmental toxicants, chemical substances and their metabolites, which enabled cells to employ the adaptive mechanisms and accelerated tumor progression as well [11]. Previous studies revealed that, B[a]P affected the extracellular chemical microenvironment, thereby interfering with body homeostasis, and promote the occurrence of HCC [12, 13]. Moreover, B[a]P also participated in the regulation of key signaling pathways, and promoted neovascularization, invasion, and metastasis of HCC [14, 15]. However, more in-depth and diverse HCC biological behaviors affected by B[a]P, and the molecular mechanisms involved in, remains largely uninvestigated.

In our present study, after long-term exposure of low dose of B[a]P, HCC cells acquired the ability of mutidrug resistance (MDR). For the molecular mechanisms, B[a]P activated a key molecule, glucose-regulated protein 75 (GRP75), which then induced a modification of apoptosis-related proteome. Among them, we identified the X-linked inhibitor of apoptosis protein (XIAP) as a key downstream factor regulated by GRP75. Briefly, in B[a]P treated HCC cells, GRP75 activated XIAP via the phosphorylation at Ser87 site. XIAP then inhibited the phosphorylation levels of caspase-9 (an apoptosis initiator) at Thr125, Ser196 and Ser144 sites. Such prevention effect blocked the activation of the key apoptosis executor, caspase-3, thereby repressed the apoptosis process in HCC cells. The long-term exposure of B[a]P promoted the continuous occurrence of the above process, and the anti-apoptosis abilities of HCC cells were gradually enhanced, ultimately leading to obtain the MDR characteristics.

## Materials and methods

### Establishment of B[a]P-induced MDR and intervention HCC cell models

Two human HCC cell lines, HepG2 and HuH7, were obtained and short tandem repeat identified by KeyGen Co. Ltd (Nanjing, China). Cells were cultured in Dulbecco’s Modified Eagle Medium (DMEM, Life Technologies/Gibco, Grand Island, NY) with 10% fetal bovine serum (FBS) (Gibco) and 1% penicillin-streptomycin (Gibco) at 37 °C in a humidified incubator containing 5% CO_2_. After 24 hours’ subculture, when HepG2 cells adhered the wall, and restored their normal appearance, we used 0.0 or 100.0 nM B[a]P (purity > 96.0%, Sigma-Aldrich Co, Shanghai, China) to culture cells for 48 to 72 h. After the cells returned to normal growth state and grow to 80–90 % confluence, the digestion and passage were performed. The above processes constituted a culture cycle. Subsequently, after a continuous induction of approximately 30 cycles, we detected the IC_50_s of cells_,_ and calculated the value of MDR cells in comparison to its parental sensitive cells, then get the drug resistance index (RI). When the RI value greater than 2, indicating such cells reached a relative high drug resistance level [16]. After then, the cells were stably passaged and cultured in fresh DMEM medium or which containing 100.0 nM B[a]P (alternating the process of cultivation with different media at one-generation intervals). Before each experiment, we first determine whether the cells are resistant to drugs (RI > 2), if so, we then proceed to the subsequent experiments, otherwise continue to apply 100.0 nM B[a]P for treatment until it reaches resistance levels. For the intervention cell model, when Huh7 cells were subcultured and return to normal adherent growth, cells were pretreated with 0.0 or 20.0 μM 3,4-dihydroxycinnamic acid (caffeic acid, CaA, purity ≥ 98.0%, Sigma-Aldrich Co) for 6 h, followed by adding 100.0 nM B[a]P for continuing culture, the later steps were similar to the construction process of HepG2 cells. The cultivation process was defined as a cycle, and repeated for nearly 30 times.

### Establishment of tumor xenograft models and intervention in nude mice

The animal experiments were approved by the Nanjing Medical University Institutional Animal Care and Use Committee (IACUC-2209053). BALB/c nude mice were obtained from the SLRC Laboratory Animal Center (Shanghai, China), and housed in a specific pathogen-free (SPF) condition with a constant temperature, humidity and a 12 h light-dark cycle, and had free access to water and chow. For the xenografts, a total of 2 × 10^6^ cells were suspended in 100.0 μl matrigel, then subcutaneously injected into the right armpit of mice for 2 weeks. Mice were randomized into 6 groups for treatment with CaA (10.0 mg/kg/twice per week, intraperitoneally [17]), cisplatin (CDDP, purity ≥ 99.99%, Sigma-Aldrich; 4.0 mg/kg/twice per week, intraperitoneally [18]), sorafenib (purity > 99.89%, Selleckchem, Shanghai, China; 30.0 mg/kg/day, gavage [19]), or combined with each other (*n* = 5). When carried out this step, the researchers used the drugs with the marked number to achieve the purpose of blinding. Tumor volumes were estimated using the formula: V = ½ (width^2^ × length). Finally, all mice were euthanized after 16 days simultaneously, and the tumor tissues were subjected to further investigation.

### Bioinformatics analysis

The microarray raw dataset of GSE36244 was downloaded from the Gene Expression Omnibus database (GEO, https://www.ncbi.nlm.nih.gov/geo/). The dataset originated from a study which characterized the transcriptomic response of HCC cells after exposure to B[a]P. After the normalization of data, differentially expressed genes (DEGs) were identified by using R software. The screening criteria for DEGs were: “*p* < 0.05 and |logFC | > 0.5”. Gene Ontology (GO) and Kyoto Encylopaedia of Genes and Genomes (KEGG) enrichment analysis were conducted to reveal the biological functions and characteristics via the Database for Annotation, Visualization and Integrated Discovery database (DAVID, http://david.ncifcrf.gov). Gene Set Enrichment Analysis (GSEA) analysis was conducted to identify the difference of the pathways by R language. The Search Tool for the Retrieval of Interacting Genes database (STRING, https://string-db.org) was applied to map the protein-protein interaction (PPI) network of GRP75 and its neighboring molecules. Gene expression data of liver hepatocellular carcinoma (LIHC) cohort was obtained from TCGA database via UCSC Xena online platform (https://xena.ucsc.edu).

### Molecular biology experiments

Cell viability assays and calculation of the 50% inhibitory dose (IC_50_) were conducted to evaluate the MDR characteristic. Flow cytometry and TdT-mediated dUTP nick end labeling (TUNEL) staining were applied to determine the apoptosis levels. The apoptosis phospho antibody array analysis was employed to evaluate the functions of GRP75 in phosphorylation modification of apoptosis-related proteome. Quantitative real-time polymerase chain reaction (qPCR), western blot, and immunohistochemistry (IHC) were used for evaluating mRNA and protein expression. Cell transfection assay was used to explore the genes’ functions. Caspase-9 and caspase-3 activity detections were used to analyse their activities. Detailed information of these methods was listed item by item in the supplementary materials.

### Statistical analysis

Statistical analysis was performed by using GraphPad Prism (version 9.0.0 for Windows; San Diego, California, USA; www.graphpad.com). Statistical significance was determined using a two-tailed Student’s t-test, one-way analysis of variance (ANOVA) followed by Dunnett’s t test, or two-way analysis of variance followed by Sidak’s multiple comparisons test. Survival analyses were evaluated by Kaplan-Meier curves and the log-rank (Mantel-Cox) test. Data were presented as mean ± standard deviation (SD, *n* = 3). Differences were considered significant when *p* < 0.05.

## Results

### Long-term exposure of HepG2 cells to low dose B[a]P caused MDR

**Fig. 1:**
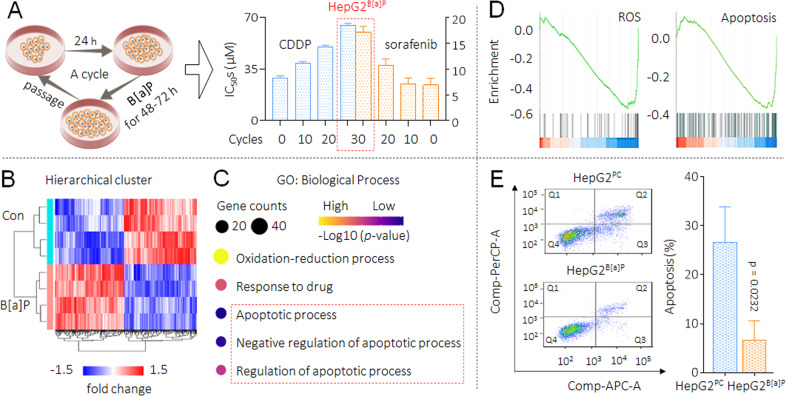
MDR caused by B[a]P, and the key biological process involved. **A**, left The flow chart for the establishment of B[a]P-induced MDR cell model in HepG2 cells. **A**, right The cell viabilities were determined in triplicate, and the IC_50_s were calculated. **B** A heat-map of the DEGs with *p* < 0.05 and |logFC | > 0.5 in GSE36244 dataset. **C** GO biological process enrichment analysis of DEGs. **D** Enrichment plots from GSEA showing the enrichments of ROS and apoptosis-related genes in the DEGs group. **E** Flow cytometric analyses of the percentage of cells in apoptosis under the treatment of 10.0 μM cisplatin (Q1: necrotic cells, Q2: late apoptosis, Q3: early apoptosis, Q4: living cells). From the histograms, the proportion of cells in total apoptosis (Q2 + Q3) was statistically analyzed.

Firstly, we constructed a cell model by long-term treatment of HepG2 cells with low doses of B[a]P. Researches confirm that the plasma B[a]P concentration in the exposed population was approximately 100.0 nM [20]. Another study revealed that after cultured with 100.0 nM B[a]P for up to 1 month, the growth rates of HCC cells were unaffected, even the migration and invasion were enhanced [14]. So, we treated HepG2 cells with various concentrations, and found that 100.0 nM B[a]P had no obvious inhibitory effect on cell viability (Supplementary Fig. S1). On the basis, we chose B[a]P at the concentration of 100.0 nM to sustained exposure. Here with the extension of processing time, the IC_50_s of CDDP and sorafenib to B[a]P-treated HepG2 cells were increased in a time-dependent manner. The exposure process continued for 30 cycles (approximately 4 months) until such cells (marked as HepG2^B[a]P^) reached a highly drug resistance level (drug resistance index value greater than 2 [16]). However, there is no significant difference in the sensitivity of HepG2 and passage control HepG2 cells (marked as HepG2^PC^) to CDDP and sorafenib (Fig. 1A and Supplementary Fig. S2). These results indicated that, after long-term B[a]P exposure, HCC cells obtained MDR phenotype.

### Revealing the key biological process in B[a]P-treated HepG2 cells

We then downloaded a microarray dataset GSE36244 from GEO database, and analyzed the DEGs between B[a]P-treated and medium control HepG2 group. A total of 230 significant DEGs were identified, and the heat map of cluster analysis was shown in Fig. 1B. Next, we performed a GO enrichment analysis and found that, oxidation-reduction, response to drug, and apoptosis-related regulation rounded out the top five biological processes (Fig. 1C and Supplementary Fig. S3). For oxidation-reduction, it has been well documented that, in B[a]P-exposed cells, intracellular antioxidant systems were activated in parallel with increased ROS levels [21]. We then conducted gene set enrichment analysis (GSEA) and revealed that the DEGs were mainly enriched in ROS and apoptosis-related pathways, and showed negative regulation trends. Namely, the generation of reactive oxygen species was reduced and apoptosis was inhibited (Fig. 1D). Apoptosis is one of the important mechanisms of CDDP and sorafenib in inhibiting HCC [22, 23], therefore, we further carried out a flow cytometry detection and found that, under the cisplatin treatment, the apoptosis level was lower in HepG2^B[a]P^ compared with HepG2^PC^ cells (Fig. 1E). These results indicated that, long-term B[a]P exposure continuously induced HepG2 cells to employ the adaptive mechanisms for survival (antiapoptosis ability), leading to the acquirement of MDR ability.

### Revealing a key anti-apoptotic molecule in HepG2 and HepG2^B[a]P^ cells

**Fig. 2:**
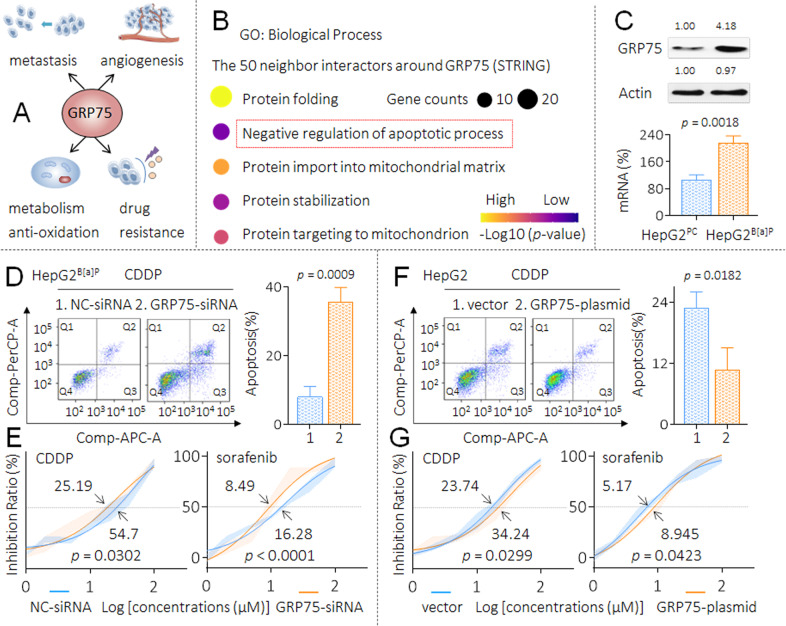
Revealing a key anti-apoptotic molecule in HepG2 and HepG2^B[a]P^ cells. **A** Schematic diagram of the biological functions of GRP75 in HCC. **B** The top 5 GO biological processes of GRP75 and the 50 most frequently altered neighbor interactors around it. **C** Triplicate qPCR and western blot analysis of the mRNA and protein levels of GRP75 in HepG2^PC^ and HepG2^B[a]P^ cells. **D** and **F** After transfection, flow cytometric analyses of the percentage of cells in apoptosis under the treatment of 10.0 μM cisplatin. **E** and **G** Transfected cells were treated with different concentrations of CDDP (0.0 to 100.0 μM) and sorafenib (0.0 to 100.0 μM) for 24 h, cell viability was determined in triplicate, and IC_50_s were calculated.

In our previous studies, we revealed a molecule, GRP75 (a member of the heat shock proteins family), which functions in multiple processes of HCC, including metastasis, angiogenesis, metabolism and antioxidation; importantly, it also plays a key role in the MDR of HCC cells [24–26] (Fig. 2A). Here, we first employed a STRING database to further predict the downstream signal transduction mechanisms regulated by GRP75. As shown in Supplementary Fig. S4, with GRP75 as the central molecule, a protein-protein interaction network was generated for the 50 most frequently altered neighbor interactors. Next, we performed a GO enrichment analysis, and found that the negative regulation of the apoptotic process was one of the most critical biological processes regulated by GRP75 (Fig. 2B and Supplementary Fig. S5). Based on the above-mentioned evidence, we speculated there might be a relationship between GRP75 and anti-apoptosis/MDR ability in HCC cells under the B[a]P treatment. Here, as shown in Fig. 2C, the expression of GRP75 was significantly higher in HepG2^B[a]P^ than that in HepG2^PC^ cells. Furthermore, in HepG2^B[a]P^ cells, compared with NC-siRNA transfected group, the apoptosis level was significantly higher in GRP75-siRNA transfected group under the cisplatin treatment (Fig. 2D). The IC_50_s of CDDP and sorafenib were markedly decreased after the knockdown of GRP75 (Fig. 2E). We then forced expression of GRP75 in HepG2 cells and found that, compared with vector-transfected group, the apoptosis level was significantly lower in GRP75-plasmid transfected group under the cisplatin treatment (Fig. 2F). The IC_50_s of CDDP and sorafenib were markedly increased after the overexpression of GRP75 (Fig. 2G). These results indicated GRP75 played an important role in maintaining the anti-apoptosis/MDR ability in HepG2^B[a]P^ cells. We further put forward two thought-provoking questions. (1) During the HCC cells long-term exposed to B[a]P, whether GRP75 was involved in the process of acquiring the anti-apoptosis ability and ultimately leading to obtain the MDR characteristics. (2) The potential mechanisms of GRP75 in the abovementioned process.

### Involvement of GRP75 in B[a]P-induced MDR in HCC cells

**Fig. 3:**
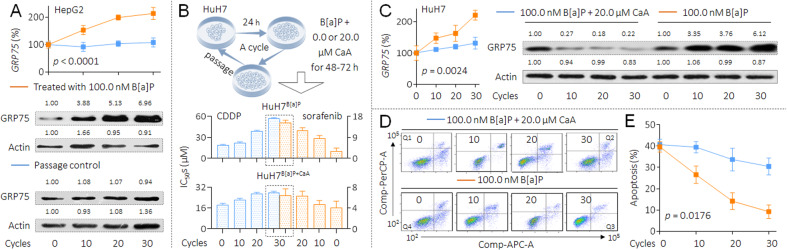
Involvement of GRP75 in B[a]P-induced MDR in HCC cells. **A** Triplicate qPCR and western blot analysis of the levels of GRP75 mRNA and protein in HepG2^PC^ and HepG2^B[a]P^ cells. **B**, up The flow chart for the establishment of B[a]P-induced MDR and intervention cell model in HuH7 cells. (B, bottom) The cell viabilities were determined in triplicate, and the IC_50_s were calculated. **C** Triplicate qPCR (left) and western blot (right) analysis of the levels of GRP75 mRNA and protein in HuH7^B[a]P^ and HuH7^B[a]P+CaA^ cells. **D** and **E** Flow cytometric analyses of the percentage of cells in apoptosis under the treatment of 10.0 μM cisplatin.

In HepG2 cells, with increased time of exposure to B[a]P, we found more increased expression of GRP75 and the elevated activities of NF-κB (an upstream transcriptional regulator of GRP75 [26]), while no such changes were observed in passage control cells (Fig. 3A and Supplementary Fig. S6). Next, we constructed another cell model by long-term B[a]P exposure in combination with GRP75 inhibition. MKT-077 (C_21_H_22_ClN_3_OS_2_) is one of the classic chemical inhibitors of GRP75, which works by eliminating the interactions between GRP75 and p53, but does not alter the expression of GRP75 [27]. However, the HCC patients in Southeast Asia (e.g. China and Japan) harbor a high frequency (up to 60%) of p53 mutations [28]. So here we used another GRP75 inhibitor, 3,4-dihydroxycinnamic acid (caffeic acid, CaA), which exerted a dual inhibitory effect on GRP75 by both transcriptional and post-transcriptional levels (Supplementary Fig. S7) [26]. HuH7 cells (with mutant p53) were long-term treated with low doses (100.0 nM) of B[a]P, in the presence or absence of 20.0 μM CaA. The doses selection of these two chemicals were based on our previous study [26] and on the Supplementary Fig. S8. With the extension of processing time, the IC_50_s of CDDP and sorafenib to B[a]P alone treated cells were increased in a time-dependent manner. The exposure process continued for 30 cycles until such cells (marked as HuH7^B[a]P^) reached a highly drug resistance level. However, the upward trend of the IC_50_s (CDDP and sorafenib) was markedly inhibited in cells co-treated by B[a]P and CaA (marked as HuH7^PC^, Fig. 3B and Supplementary Fig. S9). In this process, the expression of GRP75 in B[a]P alone treated cells was gradually increased, while in B[a]P combined CaA treated cells, such increase trend was also significantly inhibited. Indeed, CaA suppressed the expression of GRP75 mRNA and protein expression throughout the whole exposure process (Fig. 3C). Furthermore, with increased time of exposure to B[a]P alone, we found more decreased apoptosis levels under the cisplatin treatment. This downward trend of apoptosis was also markedly inhibited in cells treated by B[a]P plus CaA (Figs. 3D and 3E). These results suggested that, during the long-term exposed to B[a]P, GRP75 played an important role in inducing HCC cells to enhance the anti-apoptosis ability, ultimately leading to obtain the MDR characteristics.

### Potential mechanisms of GRP75 in B[a]P-induced MDR in HCC cells

**Fig. 4:**
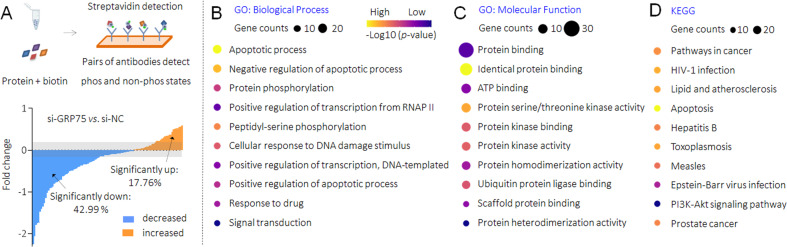
GRP75-induced phosphorylation of apoptosis-related proteome modification in HCC cells. **A** The apoptosis phospho antibody array analysis of the changes of phospho-proteins following GRP75 knockdown in HepG2^B[a]P^ cells. Upregulation of phosphorylation by more than 1.2-fold is shown in orange, and downregulation of more than 1.2-fold is shown in blue. **B**–**D** GO and KEGG pathway enrichment analyses of the differential proteins. The size of the circle indicated the number of genes enriched, and different color shades indicated the size of *p* value.

**Fig. 5:**
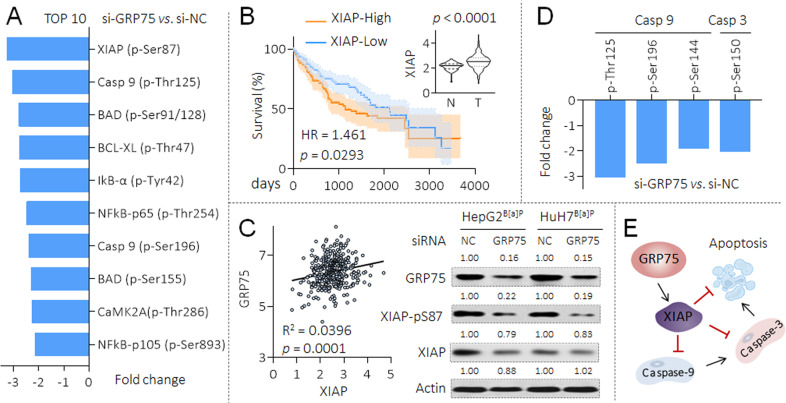
Potential mechanisms of GRP75 in B[a]P-induced MDR in HCC cells. **A** The top 10 proteins with the most significant phosphorylation changes in the phospho antibody array, the data of each phosphorylation site levels exhibited was the mean of two biological replicates. **B** Expression levels in tumor/para-cancerous tissues and prognostic significance of XIAP protein based on the TCGA. (**C**, left) The correlation analysis between GRP75 and XIAP based on TCGA. (**C**, right) After the transfection with si-NC or si-GRP75, western blot analysis of the expressions of GRP75, XIAP(p-S87), and XIAP. **D** The phosphorylation levels of caspase-9 and caspase-3 with different sites in the phospho array. **E** A mechanism map speculating the potential mechanisms involved in GRP75-induced anti-apoptosis.

We then further investigated the potential mechanisms of GRP75-induced anti-apoptotic process in B[a]P-caused MDR HCC cells. As mentioned above, mutations of *p53* gene is a common event in HCC [28], so GRP75 also promotes HCC via several other ways, as synergistic effects with CD151, human telomerase reverse transcriptase, and heterogeneous nuclear ribonucleoprotein K [29, 30]. Our latest study revealed that GRP75 was involved in the phosphorylated modification of cancer-associated proteome in HCC [26]. Therefore, we transfected HepG2^B[a]P^ cells with NC-siRNA or GRP75-siRNA, and performed an apoptosis phospho antibody array to identify the spectrum of proteins (247 antibodies, including 107 phosphorylation sites) with a change of more than 20% in phosphorylation between the two groups. As shown in Fig. 4A and Supplementary Table S1, a total of 65 proteins (containing different phosphorylation sites, of which 46 were down-regulated, accounting for 42.99%; 19 were up-regulated, accounting for 17.76%) were significantly changed. For the differential proteins, we conducted the GO and KEGG enrichment analysis, confirmed the functions of GRP75 in regulating the apoptosis process (Figs. 4B to 4D). We then listed the top ten proteins which with the most significantly changed trends, among them, found XIAP ranked the highest. Interestingly, our results also showed, the knockdown of GRP75 inhibited the phosphorylation levels of some crucial phosphorylation sites of NF-κB, suggesting there might be a positive feed-back loop between GRP75 and NF-κB (Fig. 5A). Next, based on the TCGA database, the expression of XIAP was higher in HCC than that in para-cancerous tissues, and was associated with poor prognosis (Fig. 5B). We also found that there was a positive correlation between the expressions of GRP75 and XIAP in HCC tissues, and that, knockdown of GRP75 decreased the phosphorylation of XIAP at Ser87 site (a classical phosphorylation site, enhancing the stability of XIAP) and its mRNA levels in both HepG2^B[a]P^ and HuH7^B[a]P^ cells (Fig. 5C and Supplementary Fig. S10). XIAP is one of the key inhibitors of the apoptosis proteins family (IAP), which blocked the activities of caspase-9 and caspase-3, sparing cells from apoptosis caused by various stimuli [31]. Then we analyzed the change trends in phosphorylation levels of caspase-9 and caspase-3 with different sites and found that the expression of phosphorylated caspase-9 (p-Casp9) Thr125, p-Casp9 Ser196/144, and p-Casp3 Ser150 (four classical activity inhibition sites [32]) were all showed varying degrees of decline in GRP75-siRNA transfected HepG2^B[a]P^ cells (Fig. 5D). These results indicated that, GRP75 participated in B[a]P-induced MDR of HCC cells via the phosphorylated regulation of XIAP, and then blocked the caspase cascade activation, leading to the anti-apoptosis signaling (Fig. 5E).

### Verification of the results in vitro and in vivo

**Fig. 6:**
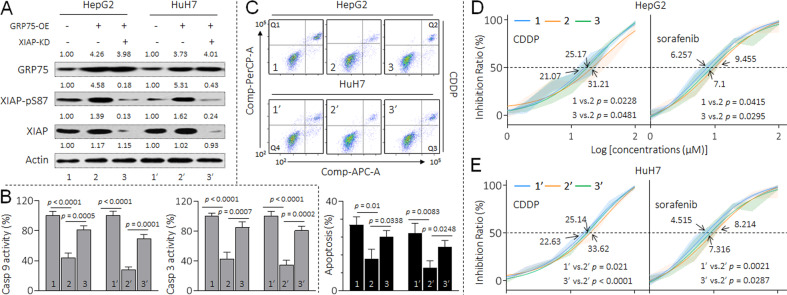
Verification of the results in vitro. HepG2 and HuH7 cells were transfected with GRP75-plasmid in the presence or absence of XIAP-siRNA. **A** Western blot analysis of the expressions of GRP75, XIAP(p-S87), and XIAP. **B** Relative caspase-9/3 activities. **C** Flow cytometric analyses of the percentage of cells in apoptosis under the treatment of 10.0 μM cisplatin. **D** and **E** Cell viability was determined in triplicate, and the IC_50_s were calculated.

To validate the aforementioned analysis in vitro, we co-transfected GRP75-plasmid and XIAP-siRNA in HCC cells. As shown in Fig. 6A, after overexpression GRP75 in both HepG2 and HuH7 cells, the phosphorylation of XIAP at Ser87 site were increased; however, knockdown of XIAP attenuated such phenomenon. Meanwhile, the forced expression of GRP75 declined the caspase-9 and caspase-3 activities and inhibited the apoptosis under the treatment of cisplatin; nevertheless, when XIAP was knocked down, these effects were blocked (Figs. 6B and 6C, Supplementary Table S2). Likewise, the IC_50_s of CDDP and sorafenib of HepG2 and HuH7 cells were markedly elevated after the transfection of GRP75 plasmid, but were also significantly reversed by the co-transfection of XIAP-siRNA (Figs. 6D and 6E).

**Fig. 7:**
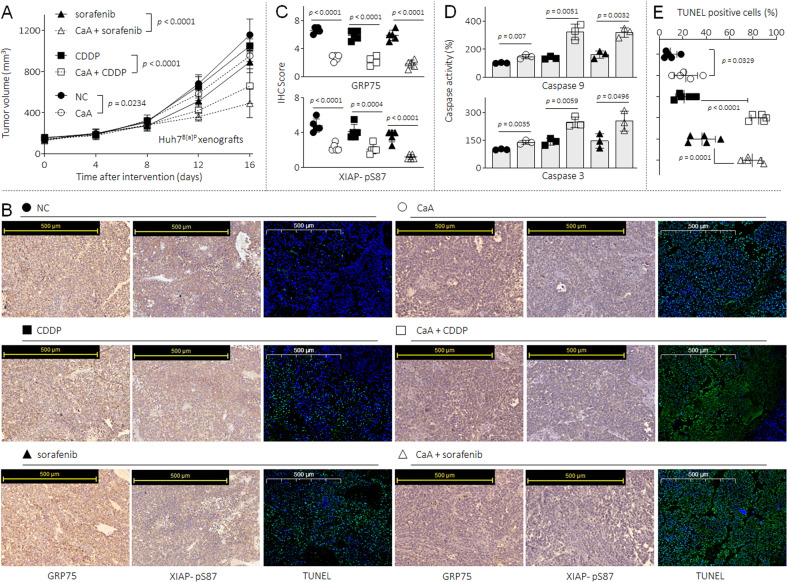
Verification of the results in vivo. The HuH7^B[a]P^ cell xenografts were treated with CaA, CDDP or sorafenib alone, or treated with CaA combined with CDDP or sorafenib. **A** The volumes of xenografts tumors. **B** and **C** IHC staining of the GRP75 and XIAP (p-Ser87). **D** Relative caspase-9/3 activities in the xenografts tissues. **B** and **E** TUNEL staining and quantitative analysis.

Finally, we validated our results in vivo via establishing a tumor xenograft and intervention model in nude mice. The HuH7 ^B[a]P^ cell xenograft-bearing mice were divided into 6 groups randomly: NC, CaA, CDDP, sorafenib, CaA plus CDDP, and CaA plus sorafenib. As shown in Fig. 7A, CaA, CDDP or sorafenib treatment alone could inhibit the tumor growth; when mice were treated with CDDP or sorafenib in combination with CaA, such inhibitory effects were significantly enhanced. In CaA, CaA plus CDDP, and CaA plus sorafenib treated group, the expressions of GRP75, p-XIAP(Ser87) and the activities of NF-κB were markedly decreased compared with NC, CDDP, or sorafenib treated group, respectively (Figs. 7B, 7C and Supplementary Fig. S11). Moreover, compared with NC group, the caspase-9 and caspase-3 activities and TUNEL-positive cells were increased in CaA, CDDP, and sorafenib-treated group. Furthermore, such apoptotic markers were significantly elevated in CaA plus CDDP and CaA plus sorafenib treated group compared with CDDP or sorafenib treated group, respectively (Figs. 7D and 7E, Supplementary Table S3). Collectively, these results suggested that, GRP75 activated XIAP, which inhibited the activations of caspase-9, and further down-regulated caspase-3, together enhanced the anti-apoptosis ability, finally leading to the acquirement and maintenance of MDR properties in HCC cells.

## Discussion

The development of tumor is always believed to result from interactions between the host and adverse environmental exposures [33]. Epidemiological studies have confirmed that environmental chemicals might be related to the high incidence rate of various tumors, via inducing the accumulation of DNA mutations, epigenetic changes and some other biological progresses [34]. Nowadays, for the effects of those environmental exposures, there has been found a deregulation of multiple signaling pathways, including disturbances in cellular metabolism, cell cycle, apoptosis, angiogenesis, or epigenetic modifications [35]. Tumor the results of a series of these genetic changes [36].

For the MDR characteristic in malignant neoplasms and many other diseases, ABC transporters plays important role, and influences severely the chemotherapy effects [37, 38]. The multi-drug resistance gene *MDR1* and its encoded P-glycoprotein (P-gp) were considered to be the most noteworthy mechanisms of MDR [39, 40]. Based on our previous and present study, we revealed an important GRP75’s upstream transcriptional regulator, NF-κB [26]. NF-κB could directly bind to the promoter region of P-gp [41]. And there were also a consensus NF-κB binding site in the first intron of the human *MDR1* gene [42]. Inhibition of NF-κB restrained the expressions of *MDR1* and P-gp levels, and led to increased apoptotic cell death in response to treatment in various tumors [43, 44]. Furthermore, NF-κB was also a key transcriptional regulator involved in B[a]P-induced angiogenesis and metastasis of HCC [14]. Therefore, we considered that, the continuous activation of NF-κB during the process of B[a]P long-term exposure may also lead to the enhancement of functions of several ABC transporters, and affect the effective dose of anti-tumor drugs, reduce the sensitivity. Apart from this, in this study, we focus on the phenotypic changes of anti-oxidant and anti-apoptotic abilities of cells caused by the continuous activation of NF-κB/GRP75. Therefore, the role of ABC transporters and the MDR characteristic changes of cells play parallel roles in the biological changes induced by B[a]P, and the synergy between them is crucial. The novel GRP75 inhibitor CaA, which inhibited the activity of GRP75 via both transcriptional and post-transcriptional modifications in HCC. The mechanisms of its action may also encompass both of these aspects, and also provide novel ideas for the design of therapeutic agents in the further study.

Here, during the process of low-dose exposure of B[a]P, normal HCC cells transformed into malignant cells with MDR characteristics, it was mainly due to the acquisition of stronger anti-apoptosis abilities. The deregulated apoptotic signaling, especially the activation of the antiapoptotic system, enables tumor cells to escape the normal death, resulting in uncontrolled proliferation, then promote tumor survival, therapeutic drug resistance and even tumor recurrence [45]. So far, nonoperative therapy including chemotherapy and targeted therapy are the main treatment for advanced HCC, and CDDP and sorafenib are important treatment options for patients [46, 47]. Mechanically, CDDP interferes with the replication and transcription process of DNA, blocking DNA synthesis and inhibiting the cancer cell cycle, followed by activation of several signal transduction pathways, which finally lead to apoptosis, thus exerting anti-tumor effects [22]. Sorafenib is a multiple-target tyrosine kinase inhibitor, capable of facilitating apoptosis, mitigating angiogenesis and suppressing tumor cell proliferation, and then extends the survival of advanced HCC patients [23].

Although the potential mechanisms of anti-apoptosis of tumor cells are still not completely defined, there were many studies have confirmed that phosphorylation regulation is one of the key reasons for the attenuation of apoptosis of tumor cells [48]. Here, we found B[a]P exposure indirectly promoted the activity of XIAP protein, a member of inhibitor of apoptosis proteins (IAPs) family, which is a group of caspase inhibitors that selectively bind and inhibit caspases-3/7/9. It could block the downstream apoptosis pathway and inhibit cell death in response to multiple stimuli via inhibiting the functions of effector caspases-3/7, blocking the cell apoptosis caused by death receptor pathway [49]. In addition, XIAP can also block the apoptosis signaling caused by the mitochondrial pathway by affecting the activity and stability of BIR domain [50]. As described before, NF-κB was an important GRP75’s upstream transcriptional regulator of GRP75, and a key transcriptional regulator involved in B[a]P-induced angiogenesis and metastasis of HCC [14]. Therefore, we speculated that, the promotion of elevated GRP75 levels by B[a]P may be due to the transcriptional activation of NF-κB. While NF-κB transcriptional activated GRP75, NF-κB could also mediate XIAP activities, they may form a NF-κB/XIAP axis then promote the cell pro-survival response [51]. In addition, XIAP is an inhibitor of apoptosis protein, and the stability is susceptible to the regulatory effects of phosphorylation, then affect its expression [52]. Therefore, in the process, the role of NF-κB in the transcriptional activation of XIAP and the phosphorylation regulation of XIAP by GRP75 may function together and affecting the expression of XIAP. As for the phosphorylation regulation, XIAP could be directly phosphorylated by PKC and that phosphorylated at Ser87 is resistant to the protein degradation, protecting cells from apoptosis-inducing stress [52]. XIAP could also bind and phosphorylated by GSK3 at Thr180, the phosphorylation is required for the activation of Wnt signaling, but is not necessary for the apoptotic pathway [53]. Here, we indicated that, in B[a]P induced MDR HCC cells, XIAP was activated by GRP75 on Ser87 site, then blocked the caspase cascade activation, leading to the anti-apoptosis signaling.

GRP75 is a member of the Hsp70 family of proteins. It is expressed in all cell types and tissues, and can perform various essential functions [54]. The expression level of GRP75 is related to muscle activity, mitochondrial activity, and biogenesis [55]. In terms of the classical mechanisms, GRP75 interacts with the tumor suppressor p53, leading to the inactivation of p53 function and regulating the apoptosis [56]. While due to the high mutation frequency of HCC patients in Asia, in addition to the classic p53-dependent pathway, GRP75 was also shown to play an irreplaceable role in promoting cancer, involving cell cycle regulation, mitochondrial function, stress response, anti-apoptosis, metabolic regulation and other important cell biological behaviors, as well as the regulatory changes of a variety of important transcription factors, kinase/ phosphatase and related downstream key proteins [29]. In our present study, we employed an apoptosis phospho antibody array to explore the role of GRP75 in regulating the proteome, and revealed some important downstream factors regulated by its phosphorylation. However, GRP75 itself is not a phosphorylated regulatory protein, so it may regulate the above biological processes via some indirect mechanisms. Based on our previous findings, we revealed a phosphoserine/threonine binding protein, 14-3-3η, which is a novel key switch-like neoplastic factor inducing the growth, angiogenesis, and MDR properties of HCC [15, 57]. Furthermore, we also found a positive correlation between the expressions of GRP75 and 14-3-3η [26]. In fact, we have also confirmed that, activated NF-κB could transcriptionally up-regulate 14-3-3η, forming a positive feed-back loop to induce/maintain the MDR phenotype in HCC [15]. Combined with the evidence, the GRP75-induced phosphorylation of cancer-related proteome modification may be mediated by 14-3-3η/NF-κB feed-back loop.

In terms of the intervention of GRP75, previous studies revealed that caffeic acid phenethyl ester (CAPE, C_17_H_16_O_4_) and CaA were both novel specific inhibitors of NF-κB activation, and that NF-κB was an upstream transcriptional regulator of GRP75 [58, 59]. In addition, they were also able to induce the disruption of the GRP75-p53 complex, leading to nuclear translocation and the activation of p53. Furthermore, in HCC, CaA also exhibited targeted intervention to GRP75 by inducing the ubiquitin-mediated degradation. Evidence indicated that, CaA inhibited the activity of GRP75 via both transcriptional and post-transcriptional modifications in HCC [26]. So here we used CaA as the inhibitor to block the GRP75 induced effects in HCC cells.

**Fig. 8:**
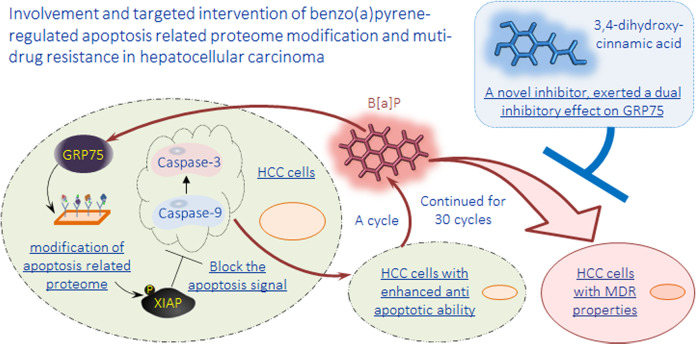
A sketch map summarizing the conclusions.

In conclusion, GRP75 played an important role in inducing/maintaining the anti-apoptosis ability and the MDR characteristics of HCC induced by low dose of B[a]P. The process was mainly mediated through the activation of GRP75, and its regulation of the phosphorylation statuses to XIAP, and several meaningful members of caspase family. We also revealed the inhibitory effect of CaA on the process. Our present study not only revealed the influence of B[a]P on the progression HCC progression, but also provided an innovative thinking for the targeted intervention (Fig. 8).

## Supplementary information

Supplementary Material

Checklist

Original Data File

## Data Availability

The data used to support the findings of this study are available from the corresponding author upon request.
