# Supplementary material for: Involvement and targeted intervention of benzo(a)pyrene-regulated apoptosis related proteome modification and muti-drug resistance in hepatocellular carcinoma
Source: Cell Death Dis. 2023 Apr 12;14(4):265. doi: 10.1038/s41419-023-05771-7 (PMC10090052; doi:10.1038/s41419-023-05771-7)
Supplement: Supplementary file 1 — Supplementary Material [file 41419_2023_5771_MOESM1_ESM.docx]

**Supplementary Material**

Involvement and targeted intervention of benzo(a)pyrene-regulated apoptosis related proteome modification and muti-drug resistance in hepatocellular carcinoma

Ye Yang ^1, *^, Ming Jin ^1, *^, Yajie Meng ^1^, Yi Dai ^2^, Shuai Chen ^2^, Yan Zhou ^2^, Yuan Li ^1, 2, 🖂^, and Liming Tang ^2, 🖂^

1. The Key Laboratory of Modern Toxicology, Ministry of Education, School of Public Health, Nanjing Medical University, Nanjing, 211166, China.
2. The Affiliated Changzhou Second People's Hospital of Nanjing Medical University, Changzhou, 213003, China.

* Ye Yang and Ming Jin contributed equally to this work

^🖂^ Corresponding author informations

Dr. Yuan Li, E-mail: liyuan@njmu.edu.cn

Dr. Liming Tang, liming_tang@sina.cn

**Inventory**

Supplementary Materials and Methods, pages 2-6.

Supplementary Table, pages 7-13.

Supplementary Figures, pages 14-19.

Supplementary References, pages 20.

**Supplementary Materials and Methods**

**Cell viabilities and calculation of the 50% inhibitory concentrations (IC_50_)**

Cell viabilities were determined by Cell Counting Kit-8 (CCK-8) (Beyotime, Shanghai, China). Briefly, cells were cultured in 96-well plates, and 10.0 μl of CCK-8 reagent was added to each well for 4 h incubation, then measured the absorbance by Infinite M200 microplate reader (Tecan, Switzerland). The IC_50_s were calculated via a graph-pad software (version 9.0.0). The inhibition ratio was determined through the dose-response equation and nonlinear regression. Sigmoidal curves were generated to obtain the IC_50_ values. As we described previously, the ordinate represented the inhibition ratio, while the abscissa represented the concentration (log), the calculation mode used was “log (inhibitor) vs. response (three parameters)” ^1^.

**Flow cytometry**

After cells were treated as indicated for 24 h, they were harvested with trypsin-EDTA, then washed twice with phosphate buffered saline (PBS) by centrifugation and fixed with 1.0 ml of ice-cold 70% ethanol overnight. The fixed cells were centrifuged, suspended in lysis buffer and incubated with RNase A for 10 min at room temperature. Cell apoptosis analyses were performed using Annexin V-PE/7-AAD Kit (Multi Sciences, Hangzhou, China) according to the manufacturer’s instruction.

**Quantitative real-time polymerase chain reaction (qRT-PCR)**

Total cellular RNA was isolated by TRIzol Reagent (Invitrogen, Carlsbad, CA), and was reverse transcribed into cDNA. qRT-PCR was performed in triplicate by using Light Cycler 96 machine (Roche Applied Science, Germany) with SYBR Green Master Mix (Vazyme Biotech, Nanjing, China). Primer sequences were as follows ^2^: *GRP75* forward, 5’-GCTGTCAATCCTGATGAGGCTGTG-3’ and reverse, 5’-CTGGCTCTTCTTGGTTGGAATAGTGG-3’; *XIAP* forward, 5’-GCTCCACGA GTCCTACTGTG-3ʹ and reverse, 5ʹ-GTTCACTGCGACAGACATCTC-3ʹ; *β-Actin* forward, 5’-GACCTGAC CTGCCGTCTA-3’ and reverse, 5’GGAGTGGGTGTCGC TGT-3’. The fold changes in expression of each gene were calculated by a comparative threshold cycle (Ct) method using the formula 2^-(ΔΔCt)^.

**Western blot**

Cells were lysed with cold RIPA lysis buffer (Beyotime) and measured with the BCA Kit (Beyotime). Samples containing 20.0 μg proteins were loaded to sodium dodecyl sulfate polyacrylamide gel electrophoresis (SDS-PAGE) for electrophoresis, and transferred to a PVDF membrane. After blocking with TBS buffer containing 5% nonfat milk, the membrane was incubated with primary antibodies and secondary antibodies according to the manufacturer's instructions. The protein levels were detected and visualized by ultra-sensitive enhanced chemiluminescent (ECL) substrate. The antibodies were purchased from the indicated sources: GRP75 (Cell Signaling, No. 3593, 1: 1000), XIAP (Cell Signaling, No. 14334, 1: 1000), Phospho-XIAP (Ser87) (Thermo Fisher, No. PA5-38349, 1: 500), and Actin (Cell Signaling, No. 4970, 1: 1000). Densitometric analysis was determined in triplicate via the Image-Pro-Plus software (version 6.0; Media Cybernetics, Georgia, USA).

**Cell transfection**

Scrambled and pcDNA-3.1-GRP75-Flag were synthesized by Generay Biotech (Shanghai, China); the negative control NC-siRNA and GRP75-siRNA were purchased from Santa Cruz Biotechnology (No. sc-37007 and No. sc-35520). The transfection process was performed following the manufacturer's instructions. Briefly, 5.0 ng/ml of plasmids or 20.0 nM of siRNAs were mixed with lipofectamine 3000 reagent (Invitrogen) in mediums containing 10% FBS without antibiotics. After 12 h of transfection, cells were cultured for another 24 h, then used in other experiments.

**NF-κB transcription factor assay**

The NF-κB activation levels were were measured by NF-κB p65 Transcription Factor Assay Kit (Abcam, Cambridge, UK) according to the manufacturer's instructions. Nuclear extracts were prepared from the cells, and added them into wells. After 1 h incubation, washed them with buffer. After that, NF-κB antibody was loaded into each well and incubated for 1 h. After washing, HRP-conjugated secondary antibody was added and incubated. Then added developing solution and incubated for 15-45 min. Finally, added stop solution and measured the absorbance with Infinite M200 microplate reader (Tecan).

**Apoptosis phospho antibody array analysis**

The phosphoprotein profiling was designed and manufactured by using the Apoptosis Phospho Antibody Array (PAP247) from Wayen Biotechnology (Shanghai, China). Samples were processed by protein extraction, lysate and marker buffer replacement, protein quantification, biotin labeling, and other operations according to the manufacturer’s recommendations. Then, array closure, incubation, protein and array hybridization, and streptavidin combined with Cy3 detection were performed. The Surescan Dx Microarray Scanner (Agilent Technologies, CA, USA) was used to scan the array. The array contained 247 antibodies, and each phosphorylation site was detected by two different types of antibodies to identify the phosphorylation status. The data for each phosphorylation site were presented as the mean of two biological replicates. GenePix Pro software (version 6.0; CA, USA) was used to read the original data ^3^. The data were shown in Supplementary Table S1. Phosphorylation levels were calculated by the equation:

$$\text{Phosphorylation ratio}\text{ }\text{=}\text{ }\frac{\text{Phosphorylated value}}{\text{unphosphorylated value}}$$

**Detection of caspase-3 and caspase-9 activities**

Caspase activities were measured by the Caspase-3 and Caspase-9 Activity Assay Kit (Beyotime), respectively. The cell and tissue samples were collected, and measured the protein concentrations. Total protein was used to quantify caspase-3/9 activity levels according to the manufacturer’s instructions ^4^. The absorbance at 405 nm was measured via Infinite M200 microplate reader (Tecan).

**Immunohistochemistry (IHC)**

The sections were mounted on silanized slides, dewaxed in xylene, dehydrated in ethanol, boiled in 0.01 M citrate buffer (pH 6.0) for 20 min in a microwave oven, and then incubated with 3% hydrogen peroxide for 5 min. The sections were washed in PBS, incubated in 10% normal bovine serum albumin for 5 min, and then incubated with the primary antibody at 4ºC overnight. The slides were then incubated with a horseradish peroxidase-conjugated secondary antibody for 30 min. The dilution ratio of the antibodies was 1: 50 according to the instructions. The samples were then visualized using diaminobenzidine, dehydrated, cleared, mounted, and photographed using a panoramic-scan digital slice scanning system (3DHISTECH Co. Ltd, Budapest, Hungary). The graphs were analyzed using Image-Pro-Plus. The quantitation of immunostaining was performed by two independent researchers who were blinded regarding the design of experiments. The immunostaining score was semi-quantified using quick-score (Q-score) based on intensity and heterogeneity as described previously ^5^.

**TdT-mediated dUTP nick end labeling (TUNEL) staining**

After slides were deparaffinized and rehydrate, they were cultivated with proteinase K working solution to cover objectives and incubated at 37 ℃ for 25 min. Then washed three times with PBS for 5 min each. Added permeabilize working solution to cover objective tissue, incubated at room temperature for 20 min, added buffer for equilibrium. After the process, followed by TUNEL reaction solution at 37 ℃ for 2 h, be sure to keep the wet box moist by adding water. Next, slides stained with 4',6-diamidino-2-phenylindole (DAPI) solution for 10 min in dark place, and washed three times with PBS, then coverslip with anti-fade mounting medium. Finally, microscopic examination was performed and images were collected via fluorescence microscope.

**Supplementary Table**

**Table S1. A list of significantly changed phosphorylated proteins in HepG2^B[a]P^ cells**

| Protein Name/Site | si-NC | | |  | si-GRP75 | | | Ratio | Fold | Trend |  |
| --- | --- | --- | --- | --- | --- | --- | --- | --- | --- | --- | --- |
|  | Pho-1 | Pho-2 | Ave. |  | Pho-1 | Pho-2 | Ave. |  |  |  |  |
| XIAP (p-Ser87) | 1.86 | 1.16 | 1.51 |  | 0.46 | 0.47 | 0.47 | 0.31 | 3.25 | ↓ | |
| Caspase 9 (p-Thr125) | 1.34 | 1.27 | 1.31 |  | 0.53 | 0.33 | 0.43 | 0.33 | 3.05 | ↓ | |
| BAD (p-Ser91/128) | 1.74 | 1.28 | 1.51 |  | 0.68 | 0.40 | 0.54 | 0.36 | 2.78 | ↓ | |
| BCL-XL (p-Thr47) | 0.75 | 0.56 | 0.66 |  | 0.25 | 0.23 | 0.24 | 0.36 | 2.75 | ↓ | |
| IkB-α (p-Tyr42) | 1.52 | 1.36 | 1.44 |  | 0.55 | 0.51 | 0.53 | 0.37 | 2.73 | ↓ | |
| NFkB-p65 (p-Thr254) | 2.17 | 1.95 | 2.06 |  | 1.00 | 0.67 | 0.84 | 0.41 | 2.47 | ↓ | |
| Caspase 9 (p-Ser196) | 0.57 | 0.44 | 0.51 |  | 0.48 | 0.34 | 0.21 | 0.42 | 2.40 | ↓ | |
| BAD (p-Ser155) | 2.15 | 2.15 | 2.15 |  | 0.87 | 1.02 | 0.95 | 0.44 | 2.28 | ↓ | |
| CaMK2A (p-Thr286) | 1.08 | 1.19 | 1.13 |  | 0.54 | 0.46 | 0.50 | 0.44 | 2.27 | ↓ | |
| NFkB-p105/p50 (p-Ser893) | 1.52 | 1.71 | 1.62 |  | 0.94 | 0.57 | 0.76 | 0.47 | 2.14 | ↓ | |

| Protein Name/Site | si-NC | | |  | si-GRP75 | | | Ratio | Fold | Trend |
| --- | --- | --- | --- | --- | --- | --- | --- | --- | --- | --- |
|  | Pho-1 | Pho-2 | Ave. |  | Pho-1 | Pho-2 | Ave. |  |  |  |
| Caspase 3 (p-Ser150) | 1.87 | 1.89 | 1.88 |  | 0.63 | 1.23 | 0.93 | 0.49 | 2.02 | ↓ |
| PTEN (p-Ser370) | 0.77 | 0.88 | 0.83 |  | 0.49 | 0.35 | 0.42 | 0.51 | 1.97 | ↓ |
| Caspase 9 (p-Ser144) | 2.55 | 2.66 | 2.61 |  | 1.57 | 1.16 | 1.37 | 0.52 | 1.91 | ↓ |
| Caspase 8 (p-Ser347) | 1.67 | 1.41 | 1.54 |  | 0.94 | 0.68 | 0.81 | 0.53 | 1.90 | ↓ |
| Lamin A/C (p-Ser392) | 0.30 | 0.41 | 0.35 |  | 0.20 | 0.18 | 0.19 | 0.54 | 1.87 | ↓ |
| ATRIP (p-Ser68/72) | 0.39 | 0.47 | 0.43 |  | 0.32 | 0.15 | 0.24 | 0.56 | 1.80 | ↓ |
| Chk1 (p-Ser345) | 1.30 | 1.27 | 1.28 |  | 0.71 | 0.74 | 0.72 | 0.56 | 1.78 | ↓ |
| P70S6K (p-Ser411) | 1.52 | 1.54 | 1.53 |  | 0.72 | 1.01 | 0.87 | 0.57 | 1.76 | ↓ |
| NFkB-p65 (p-Ser311) | 1.20 | 1.40 | 1.30 |  | 0.90 | 0.60 | 0.75 | 0.58 | 1.74 | ↓ |
| HSP90B (p-Ser254) | 1.23 | 1.01 | 1.12 |  | 0.38 | 0.92 | 0.65 | 0.58 | 1.72 | ↓ |
| BAD (p-Ser112) | 1.19 | 1.25 | 1.22 |  | 0.67 | 0.76 | 0.72 | 0.59 | 1.71 | ↓ |
| Chk1 (p-Ser317) | 1.26 | 1.67 | 1.46 |  | 0.57 | 1.23 | 0.90 | 0.62 | 1.62 | ↓ |
| Protein Name/Site | si-NC | | |  | si-GRP75 | | | Ratio | Fold | Trend |
|  | Pho-1 | Pho-2 | Ave. |  | Pho-1 | Pho-2 | Ave. |  |  |  |
| AKT1 (p-Thr72) | 0.41 | 0.46 | 0.44 |  | 0.28 | 0.25 | 0.27 | 0.62 | 1.61 | ↓ |
| Chk2 (p-Thr387) | 0.60 | 0.66 | 0.63 |  | 0.49 | 0.31 | 0.40 | 0.63 | 1.59 | ↓ |
| CDK1 (p-Thr14) | 1.40 | 1.38 | 1.39 |  | 2.28 | 2.12 | 2.20 | 1.58 | 1.58 | ↑ |
| NFkB-p100/p52 (p-Ser869) | 1.17 | 0.62 | 0.90 |  | 0.72 | 0.42 | 0.57 | 0.64 | 1.57 | ↓ |
| Chk2 (p-Thr383) | 0.91 | 0.69 | 0.80 |  | 0.90 | 1.59 | 1.24 | 1.56 | 1.56 | ↑ |
| Chk1 (p-Ser286) | 1.25 | 1.08 | 1.16 |  | 1.60 | 1.99 | 1.79 | 1.54 | 1.54 | ↑ |
| BAD (p-Ser136) | 1.25 | 1.36 | 1.30 |  | 0.75 | 0.94 | 0.85 | 0.65 | 1.54 | ↓ |
| P70S6K (p-Thr229) | 0.76 | 0.89 | 0.82 |  | 1.15 | 1.36 | 1.25 | 1.52 | 1.52 | ↑ |
| P70S6K-β (p-Ser423) | 0.82 | 0.98 | 0.90 |  | 1.30 | 1.44 | 1.37 | 1.52 | 1.52 | ↑ |
| FOXO1/3/4 (p-Thr24/32) | 0.80 | 0.87 | 0.84 |  | 1.48 | 1.05 | 1.26 | 1.51 | 1.51 | ↑ |
| PTEN (p-Ser380/Thr382/383) | 1.33 | 1.57 | 1.45 |  | 1.07 | 0.87 | 0.97 | 0.67 | 1.50 | ↓ |
| IKK-β (p-Tyr199) | 0.67 | 0.44 | 0.56 |  | 0.31 | 0.44 | 0.38 | 0.67 | 1.49 | ↓ |

| Protein Name/Site | | si-NC | | | | | |  | si-GRP75 | | | Ratio | Fold | Trend |  |
| --- | --- | --- | --- | --- | --- | --- | --- | --- | --- | --- | --- | --- | --- | --- | --- |
|  |  | Pho-1 | | Pho-2 | | Ave. | |  | Pho-1 | Pho-2 | Ave. |  |  |  |  |
| FKHR (p-Ser256) | | 1.62 | | 1.70 | | 1.66 | |  | 2.44 | 2.51 | 2.47 | 1.49 | 1.49 | ↑ | |
| B-RAF (p-Thr598) | | 0.34 | | 0.34 | | 0.34 | |  | 0.15 | 0.31 | 0.23 | 0.67 | 1.48 | ↓ | |
| IkB-ε (p-Ser22) | | 0.55 | | 0.72 | | 0.64 | |  | 0.43 | 0.44 | 0.44 | 0.69 | 1.46 | ↓ | |
| BCL-2 (p-Ser70) | | 1.71 | | 1.60 | | 1.65 | |  | 1.71 | 0.59 | 1.15 | 0.69 | 1.44 | ↓ | |
| P90RSK (p-Thr359/Ser363) | | 0.16 | | 0.26 | | 0.21 | |  | 0.43 | 0.17 | 0.30 | 1.43 | 1.43 | ↑ | |
| IKK-γ (p-Ser31) | | 0.41 | | 0.63 | | 0.52 | |  | 0.46 | 0.27 | 0.36 | 0.70 | 1.43 | ↓ | |
| 14-3-3 ζ/δ (p-Thr232) | | 0.81 | | 0.85 | | 0.83 | |  | 0.68 | 0.51 | 0.59 | 0.72 | 1.40 | ↓ | |
| p53 (p-Ser6) | | 1.78 | | 2.16 | | 1.97 | |  | 1.76 | 1.09 | 1.42 | 0.72 | 1.39 | ↓ | |
| SAPK/JNK (p-Thr183) | | 1.69 | | 1.33 | | 1.51 | |  | 2.53 | 1.62 | 2.07 | 1.37 | 1.37 | ↑ | |
| P70S6K (p-Ser424) | | 2.23 | | 1.90 | | 2.06 | |  | 2.84 | 2.82 | 2.83 | 1.37 | 1.37 | ↑ | |
| IKK-β (p-Tyr188) | | 0.87 | | 1.02 | | 0.95 | |  | 0.56 | 0.83 | 0.70 | 0.74 | 1.36 | ↓ | |
| NFkB-p105/p50 (p-Ser932) | | 0.94 | | 1.05 | | 1.00 | |  | 0.60 | 0.86 | 0.73 | 0.74 | 1.36 | ↓ | |
| Protein Name/Site | | si-NC | | | | | |  | si-GRP75 | | | Ratio | Fold | Trend |  |
|  |  | Pho-1 | | Pho-2 | | Ave. | |  | Pho-1 | Pho-2 | Ave. |  |  |  |  |
| PTEN (p-Ser380) | | 1.19 | | 1.17 | | 1.18 | |  | 0.94 | 0.82 | 0.88 | 0.75 | 1.34 | ↓ | |
| TAK1 (p-Thr184) | | 1.03 | | 1.01 | | 1.02 | |  | 1.60 | 1.14 | 1.37 | 1.34 | 1.34 | ↑ | |
| HSP27 (p-Ser15) | | 2.03 | | 2.23 | | 2.13 | |  | 1.49 | 1.69 | 1.59 | 0.75 | 1.34 | ↓ | |
| JNK1/2/3 (p-Thr183/185) | | 0.65 | | 0.71 | | 0.68 | |  | 1.03 | 0.79 | 0.91 | 1.34 | 1.34 | ↑ | |
| IKK-α (p-Thr23) | | 1.24 | | 1.37 | | 1.31 | |  | 1.16 | 0.79 | 0.98 | 0.75 | 1.34 | ↓ | |
| p44/42 MAPK (p-Tyr204) | | 1.27 | | 1.34 | | 1.31 | |  | 1.79 | 1.68 | 1.73 | 1.33 | 1.33 | ↑ | |
| NFkB-p100/p52 (p-Ser865) | | 0.66 | | 0.66 | | 0.66 | |  | 0.56 | 0.44 | 0.50 | 0.75 | 1.33 | ↓ | |
| ASK1 (p-Ser966) | | 1.47 | | 1.20 | | 1.34 | |  | 1.35 | 2.13 | 1.74 | 1.30 | 1.30 | ↑ | |
| Chk2 (p-Ser516) | | 0.82 | | 0.92 | | 0.87 | |  | 0.89 | 0.45 | 0.67 | 0.77 | 1.29 | ↓ | |
| NFkB-p65 (p-Ser536) | | 0.68 | | 0.56 | | 0.62 | |  | 0.51 | 0.46 | 0.48 | 0.78 | 1.28 | ↓ | |
| BID (p-Ser78) | 0.49 | | 0.58 | | 0.54 | |  | | 0.54 | 0.30 | 0.42 | 0.79 | 1.27 | ↓ | |
| NFkB p105/50 (p-Ser927) | 0.16 | | 0.12 | | 0.14 | |  | | 0.17 | 0.18 | 0.18 | 1.27 | 1.27 | ↑ | |

| Protein Name/Site | si-NC | | |  | si-GRP75 | | | Ratio | Fold | Trend |  |
| --- | --- | --- | --- | --- | --- | --- | --- | --- | --- | --- | --- |
|  | Pho-1 | Pho-2 | Ave. |  | Pho-1 | Pho-2 | Ave. |  |  |  |  |
| IKKα/β (p-Ser180/181) | 0.83 | 0.80 | 0.82 |  | 0.17 | 1.13 | 0.65 | 0.80 | 1.26 | ↓ | |
| IkB-β (p-Thr19) | 1.21 | 1.33 | 1.27 |  | 1.61 | 1.54 | 1.58 | 1.24 | 1.24 | ↑ | |
| Caspase 9 (p-Tyr153) | 1.22 | 0.95 | 1.08 |  | 0.81 | 0.95 | 0.88 | 0.81 | 1.24 | ↓ | |
| Chk1 (p-Ser280) | 1.67 | 2.19 | 1.93 |  | 2.16 | 2.55 | 2.36 | 1.22 | 1.22 | ↑ | |
| p53 (p-Ser392) | 0.68 | 1.00 | 0.84 |  | 0.61 | 1.44 | 1.03 | 1.22 | 1.22 | ↑ | |
| p53 (p-Ser37) | 1.26 | 1.45 | 1.36 |  | 1.99 | 1.30 | 1.64 | 1.21 | 1.21 | ↑ | |
| ASK1 (p-Ser83) | 3.20 | 2.23 | 2.72 |  | 3.08 | 1.41 | 2.24 | 0.83 | 1.21 | ↓ | |

* Note: “Pho” means the phosphorylation levels of the two parallel data of proteins.

**Table S2. Detection of caspase activities in HCC cells (absorbance, 405 nm)**

|  | caspase | NC | GRP75-OE | GRP75-OE+ XIAP-KD |
| --- | --- | --- | --- | --- |
| HepG2 | caspase 9 | 0.229 ± 0.022 | 0.101 ± 0.021 | 0.186 ± 0.019 |
|  | caspase 3 | 0.222 ± 0.015 | 0.095 ± 0.011 | 0.189 ± 0.023 |
| HuH7 | caspase 9 | 0.215 ± 0.017 | 0.062 ± 0.01 | 0.15 ± 0.013 |
|  | caspase 3 | 0.225 ± 0.02 | 0.079 ± 0.01 | 0.181 ± 0.022 |

Note: The concentration of *p*-nitroaniline (*p*NA) could be calculated via the equation (standard curve): y = 413.49X - 0.7481

**Table S3. Detection of caspase activities in xenograft (absorbance, 405 nm)**

| group | caspase 9 | caspase 3 |
| --- | --- | --- |
| NC | 0.181 ± 0.014 | 0.164 ± 0.011 |
| CaA | 0.261 ± 0.023 | 0.229 ± 0.019 |
| CDDP | 0.245 ± 0.012 | 0.234 ± 0.013 |
| CaA + CDDP | 0.574 ± 0.025 | 0.407 ± 0.021 |
| sorafenib | 0.287 ± 0.016 | 0.24 ± 0.009 |
| CaA + sorafenib | 0.567 ± 0.031 | 0.417 ±0.028 |

Note: The concentration of *p*NA could be calculated via the equation (standard curve): y = 409.71X + 0.1901

**Supplementary Figures**

**Fig. S1. The inhibitory effect of B[a]P on HepG2 cells**


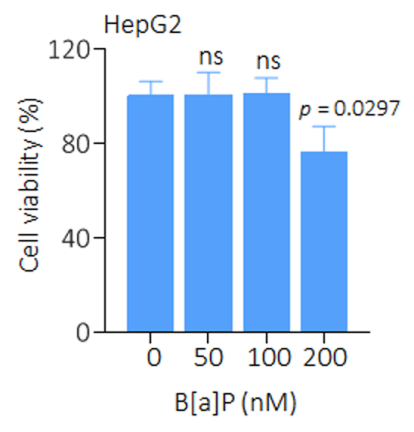


HepG2 cells were treated with various concentrations of B[a]P (0.0 to 200.0 nM) for 24 h, the cell viabilities were determined in triplicate.

**Fig. S2. The effects of B[a]P treatment on drug’s IC_50_s in HepG2 cells**


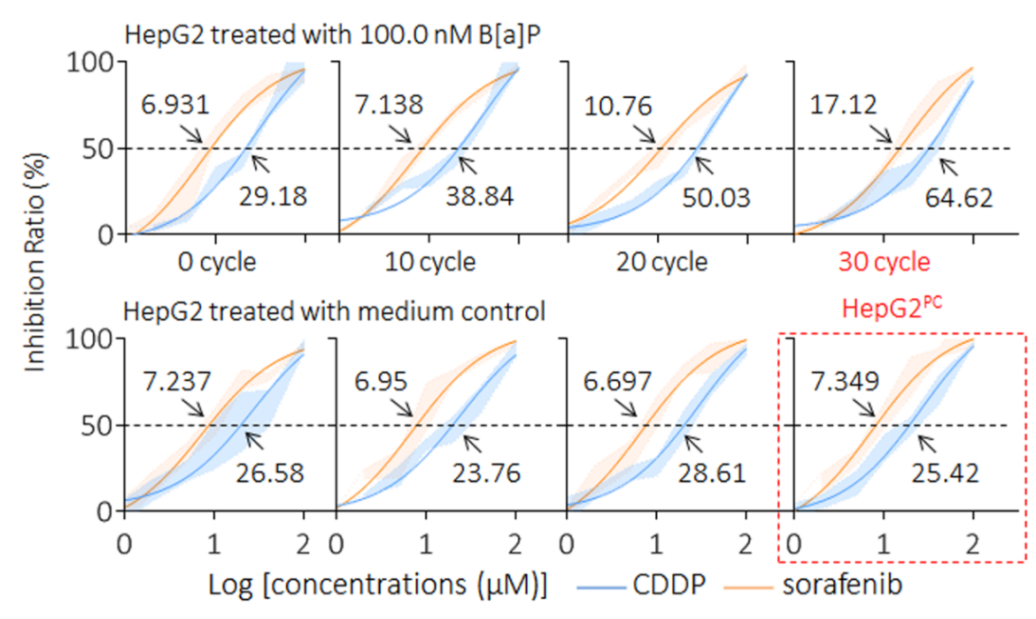


During the process of cell models construction, cell viabilities were measured every 10 cycles. Cells were treated with different concentrations of CDDP (0.0 to 100.0 μM) and sorafenib (0.0 to 100.0 μM) for 24 h at the point of different cycles, respectively. The cell viabilities were determined in triplicate, and the IC_50_s were calculated.

**Fig. S3. GO enrichment analysis of the GSE36244 dataset**


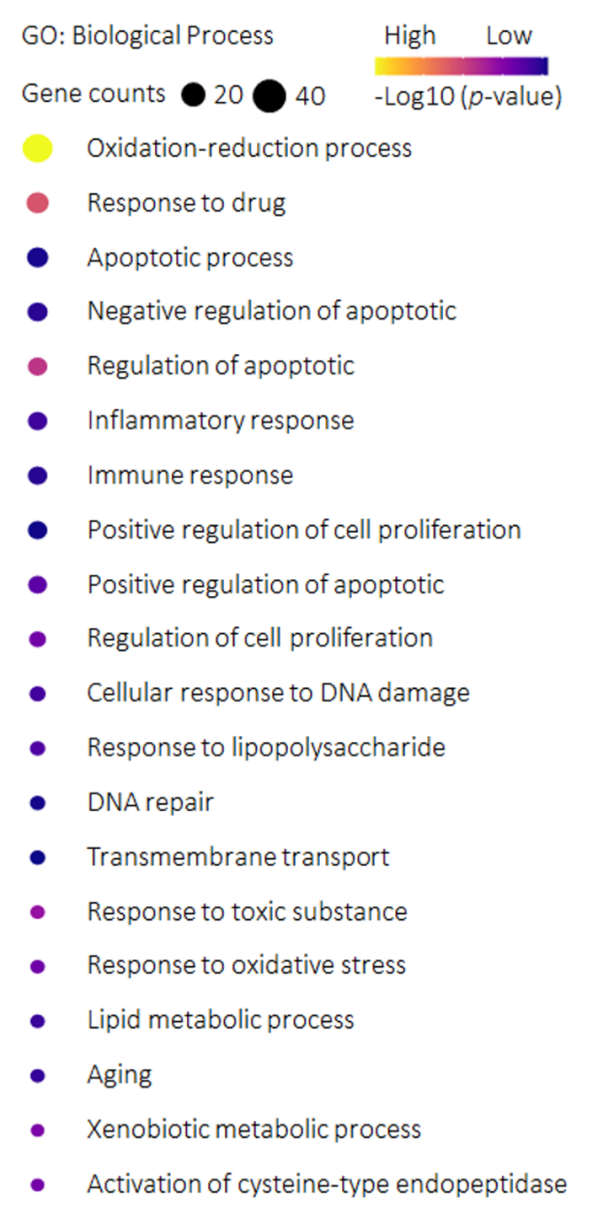


The top 20 GO biological process enrichment analysis of DEGs in GSE36244 dataset. The size of the circle indicated the number of genes enriched in the item, and different color shades indicated the size of *p* value.

**Fig. S4. The 50 most frequently altered neighbor interactors around GRP75**

**
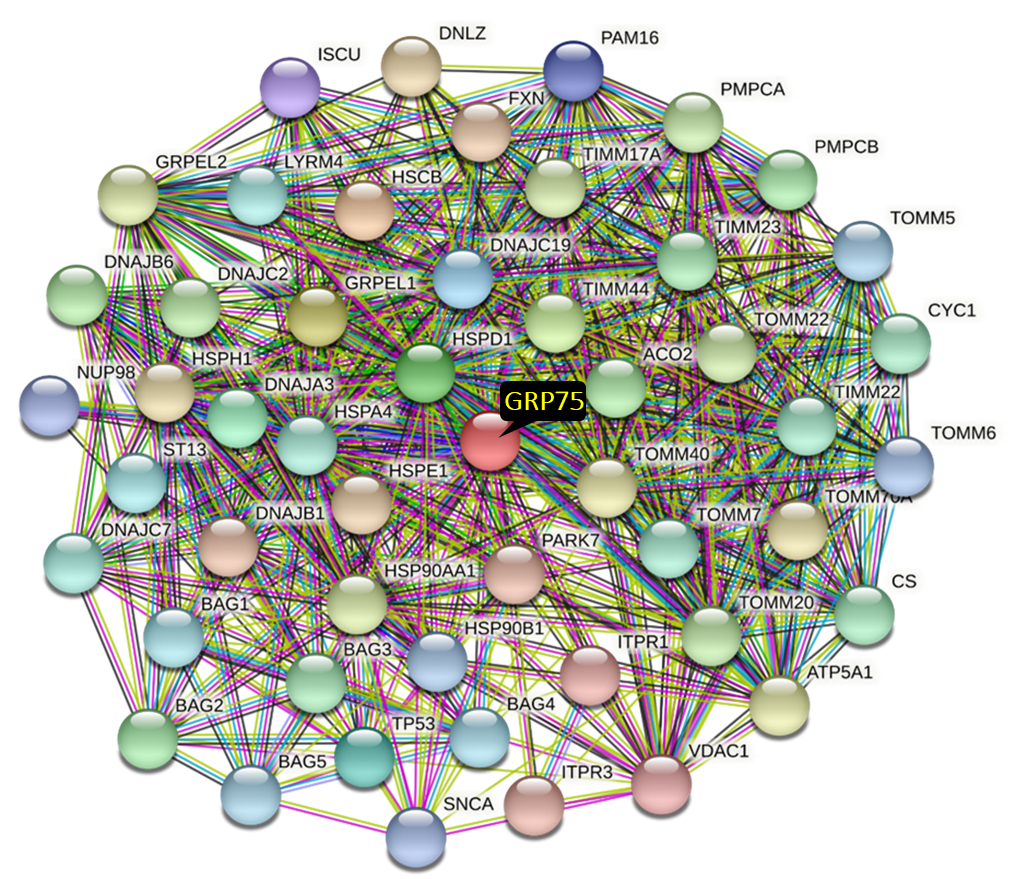
**

The protein-protein interaction (PPI) with the 50 most frequently altered neighbor interactors around GRP75 generated by STRING database.

**Fig. S5. GO enrichment analysis of the PPI around GRP75**


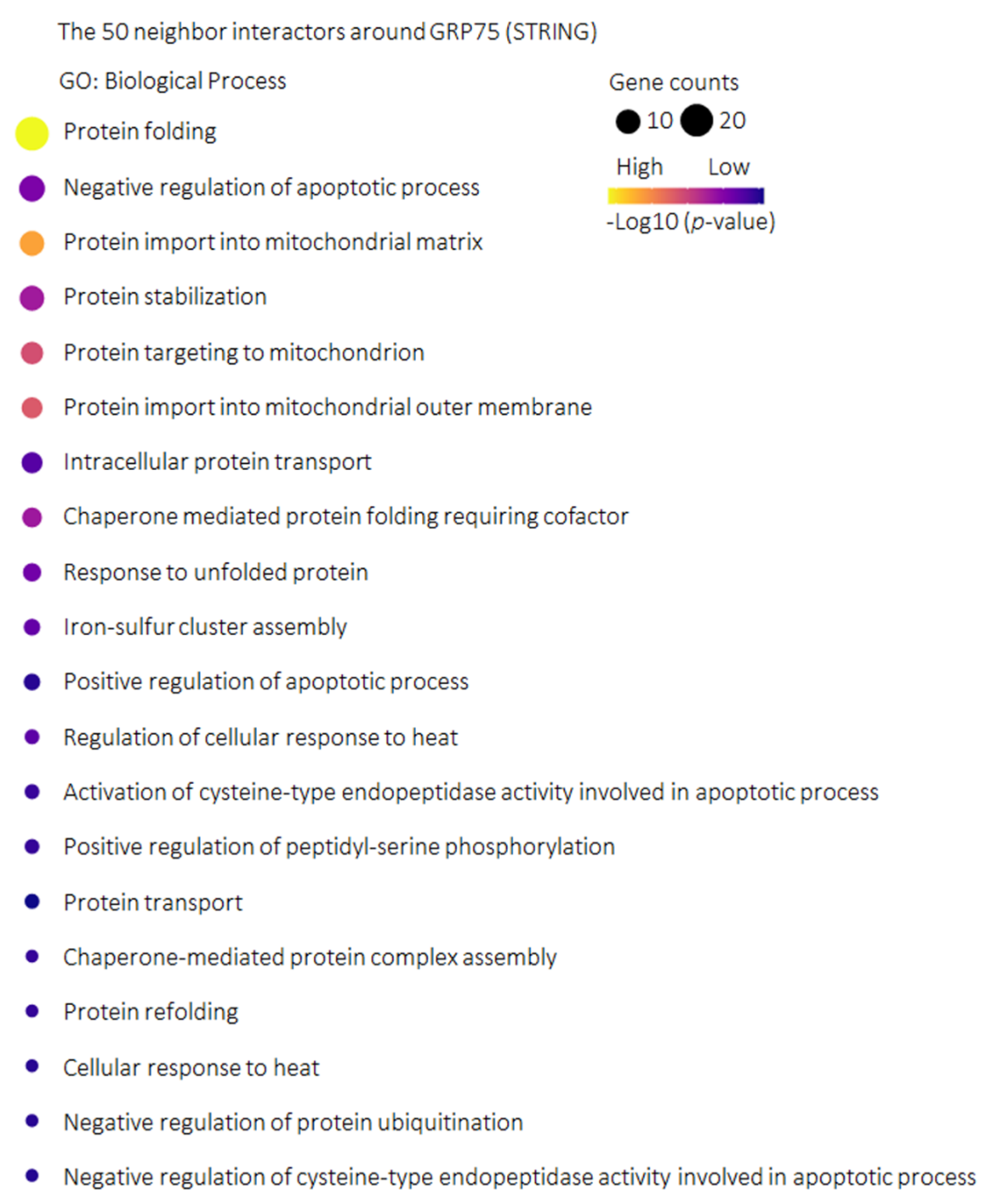


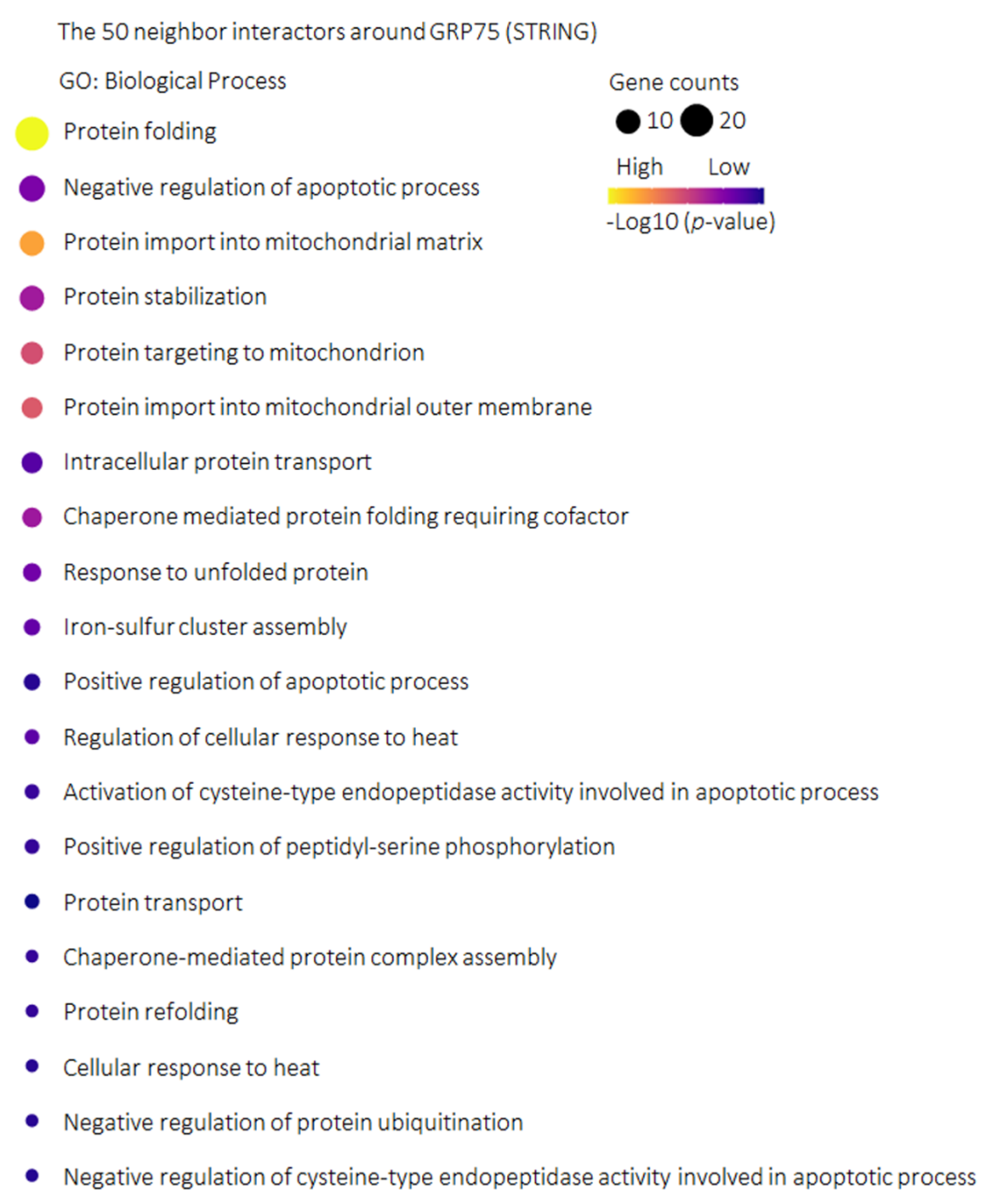


The top 20 GO biological processes of GRP75 and the 50 most frequently altered neighbor interactors around it. The size of the circle indicated the number of genes enriched, and different color shades indicated the size of *p* value.

**Fig. S6. The effects of B[a]P on NF-κB in HepG2 cells**


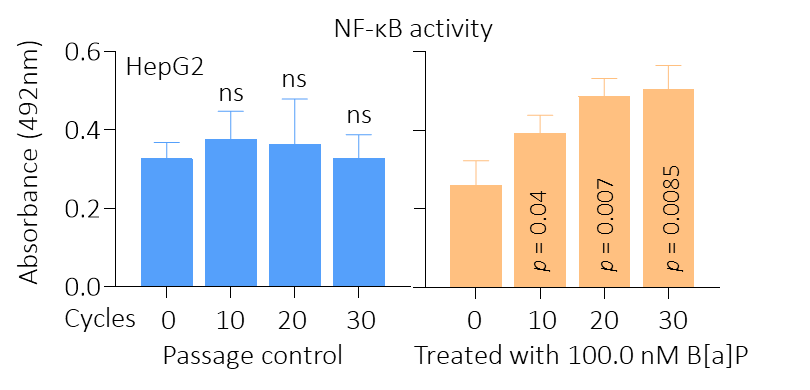


HepG2 cells were treated as described in Fig. 3A. Analysis of NF-κB activity.

**Fig. S7. Schematic diagram of the mechanism of CaA inhibiting GRP75**

**
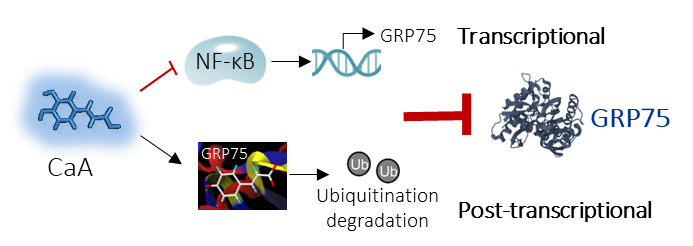
**

CaA inhibited the activity of GRP75 via both transcriptional and post-transcriptional modifications in HCC ^[3]^.

**Fig. S8. The effects of B[a]P or CaA on HuH7 cells**


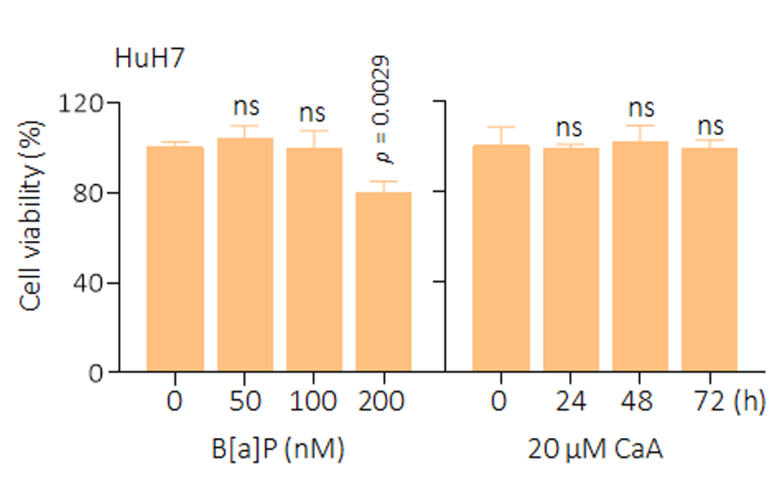


HuH7 cells were treated with B[a]P for various concentrations (0 to 200 nM), or with CaA for different time (0 to 72 h). The cell viabilities were determined in triplicate.

**Fig. S9. Effects of B[a]P and CaA treatment on drug’s IC_50_s in HuH7 cells**


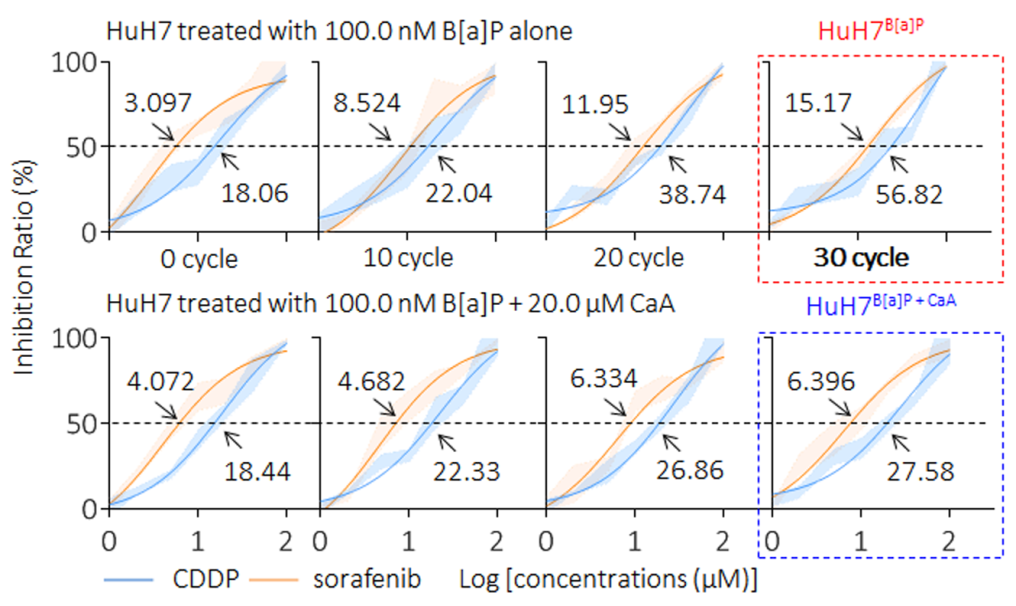


During the process of the construction of intervention cell model, cell viabilities were measured every 10 cycles. Cells were treated with different concentrations of CDDP (0 to 100 μM) or sorafenib (0 to 100 μM) for 24 h at the point of different cycles. The cell viabilities were determined in triplicate, the IC_50_s were calculated.

**Fig. S10. The effects of GRP75 on *XIAP* mRNA levels**


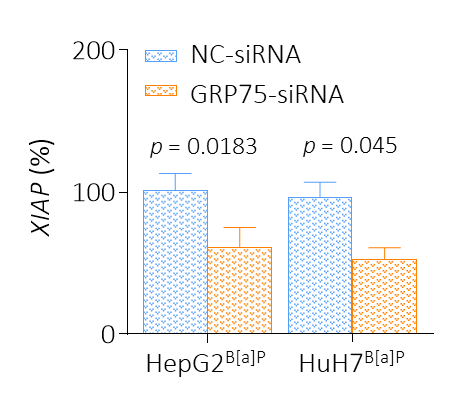


After the transfection with si-NC or si-GRP75, triplicate qPCR analysis of the mRNA levels of XIAP in HepG2^B[a]P^ and HuH7^B[a]P^ cells.

**Fig. S11. The effects of B[a]P and CaA on NF-κB in HuH7^B[a]P^ cell xenografts**


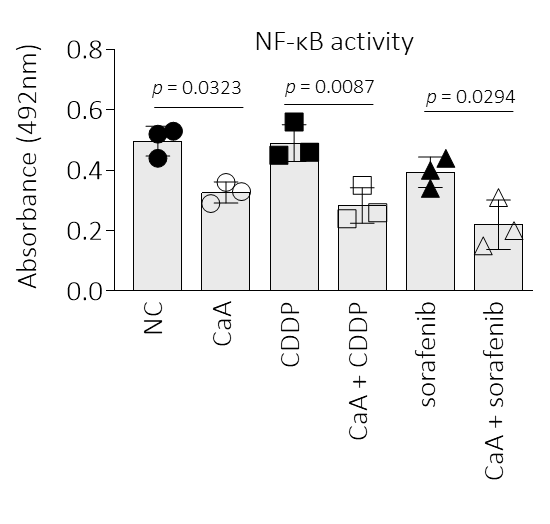


The HuH7^B[a]P^ cell xenografts were treated with CaA, CDDP or sorafenib alone, or treated with CaA combined with CDDP or sorafenib. Analysis of NF-κB activity.

**Supplementary References**

1. Qiu Y, Dai Y, Zhang C, Yang Y, Jin M, Shan W*, et al.* Arsenic trioxide reverses the chemoresistance in hepatocellular carcinoma: a targeted intervention of 14-3-3eta/NF-kappaB feedback loop. *J Exp Clin Cancer Res* 2018, **37**(1)**:** 321.

2. Jin M, Yang Y, Dai Y, Cai R, Wu L, Jiao Y*, et al.* 27-Hydroxycholesterol is a specific factor in the neoplastic microenvironment of HCC that causes MDR via GRP75 regulation of the redox balance and metabolic reprogramming. *Cell Biol Toxicol* 2022, **38**(2)**:** 311-324.

3. Yang Y, Jin M, Dai Y, Shan W, Chen S, Cai R*, et al.* Involvement and Targeted Intervention of Mortalin-Regulated Proteome Phosphorylated-Modification in Hepatocellular Carcinoma. *Front Oncol* 2021, **11:** 687871.

4. Wu YL, He Y, Shi JJ, Zheng TX, Lin XJ, Lin X. Microcystin-LR promotes necroptosis in primary mouse hepatocytes by overproducing reactive oxygen species. *Toxicol Appl Pharm* 2019, **377**.

5. Shen J, Jiang F, Yang Y, Huang G, Pu F, Liu Q*, et al.* 14-3-3eta is a novel growth-promoting and angiogenic factor in hepatocellular carcinoma. *J Hepatol* 2016, **65**(5)**:** 953-962.
