# Supplementary material for: Involvement and targeted intervention of benzo(a)pyrene-regulated apoptosis related proteome modification and muti-drug resistance in hepatocellular carcinoma
Source: Cell Death Dis. 2023 Apr 12;14(4):265. doi: 10.1038/s41419-023-05771-7 (PMC10090052; doi:10.1038/s41419-023-05771-7)

GRP75, 75 kDa

Fig. 2C

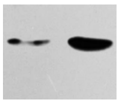

Fig. 3A

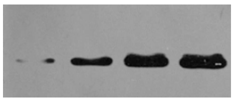

Fig. 3A

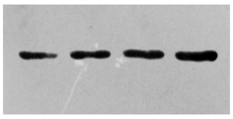

Fig. 3C

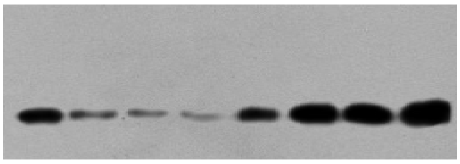

Fig. 5C

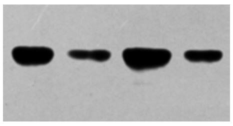

Fig. 6A

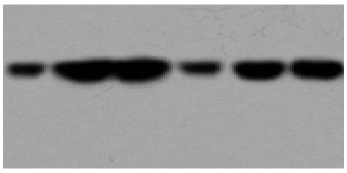

Actin, 45 kDa

Fig. 2C

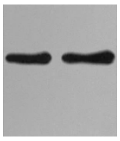

Fig. 3A

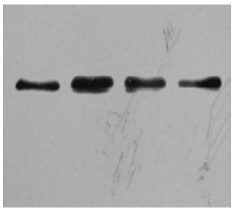

Fig. 3A

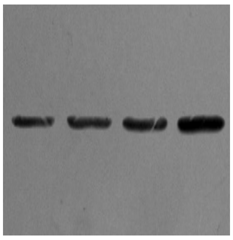

Fig. 3C

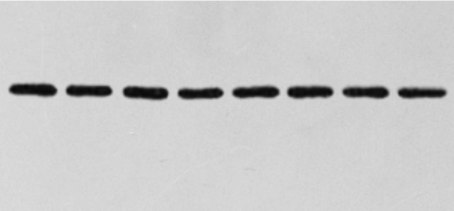

Fig. 5C

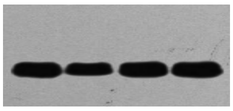

Fig. 6A

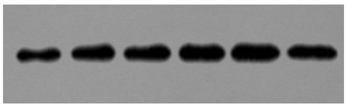

XIAP-pS87, 53 kDa

Fig. 5C

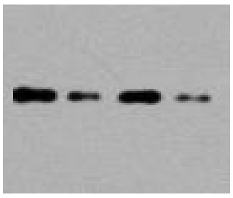

Fig. 6A

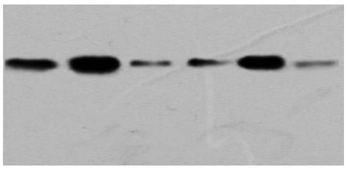

XIAP, 53 kDa

Fig. 5C

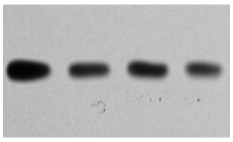

Fig. 6A

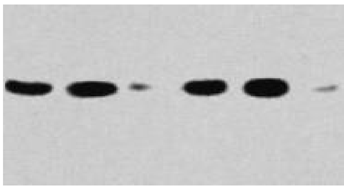

Supplement: Supplementary file 3 — Original Data File [file 41419_2023_5771_MOESM3_ESM.pdf]
